# Supplementary material for: DMRforPairs: identifying Differentially Methylated Regions between unique samples using array based methylation profiles
Source: BMC Bioinformatics. 2014 May 15;15:141. doi: 10.1186/1471-2105-15-141 (PMC4046028; doi:10.1186/1471-2105-15-141)
Supplement: Additional file 2 — DMRforPairs output for the comparison of A431-MCF7 and NA17018-NA17105. Please start from the HTML files in each folder. Available via the BMC Bioinformatics website. [file 1471-2105-15-141-S2.zip › 1394847754114233_MOESM2_ESM/NA17105M_NA17018F/significant.html]

DMRforPairs output generated on Tue Nov 19 09:30:45 2013. Identified regions were set to contain at least 4 probes with a maximum distance of 200 bp between individual probes (n=29404). Regions in which median methylation levels (M-values) between the samples differed at least |1.4| (n=4146) (=relevant) were tested for statistical significance (significant: p<0.05; multiple testing adjusted, n=28). The folowing samples were studied: NA17105-M,NA17018-F. Chromosomal positions are indicated in bp. N indicates the number of probes in a region. ID indicates the region ID. Values in the columns per sample indicate median beta values. dM indicates the largest median difference (absolute) between any of the sample pairs. p indicates uncorrected p-value from Mann-Whitney U test (n=2) or Kruskal Wallis test (n>2). p.adj denotes the multiple testing corrected p-value (method:fdr). Gene symbols of overlapping transcripts are listed for the exact region and within a margin of 10000 bp of the region.

| Thumbnail | Chr | Start | End | Links | Length | n | ID | Class | NA17105-M | NA17018-F | Gene.Symbol | dM | p | p.adj |
| --- | --- | --- | --- | --- | --- | --- | --- | --- | --- | --- | --- | --- | --- | --- |
|  | 19 | 23945472 | 23945996 | PDFSTATSENSEMBLUCSC | 525 | 8 | 21849 | island | 0.0149 | 0.91380 | RP11-255H23.2; RPSAP58 (margin: RP11-255H23.2; RPSAP58; ZNF681) | 8.75 | 1.55e-04 | 0.0296603652 |
|  | 6 | 32551749 | 32552670 | PDFSTATSENSEMBLUCSC | 922 | 17 | 34122 | gene;island | 0.0628 | 0.97607 | HLA-DRB1 (margin: HLA-DRB1) | 6.99 | 8.31e-08 | 0.0000689340 |
|  | 5 | 1594579 | 1595048 | PDFSTATSENSEMBLUCSC | 470 | 9 | 32635 | island | 0.4959 | 0.01314 | CTD-2012J19.3; SDHAP3 (margin: CTD-2012J19.3; CTD-2012J19.2; SDHAP3; RP11-43F13.1) | 6.27 | 1.65e-04 | 0.0296603652 |
|  | 6 | 31164743 | 31164994 | PDFSTATSENSEMBLUCSC | 252 | 12 | 34803 | tss;island | 0.5294 | 0.01063 | HCG27; XXbac-BPG299F13.17 (margin: HCG27; XXbac-BPG299F13.17; XXbac-BPG299F13.14) | 6.22 | 2.96e-06 | 0.0017522235 |
|  | 20 | 55841869 | 55842071 | PDFSTATSENSEMBLUCSC | 203 | 8 | 25840 | tss;island | 0.4565 | 0.01901 | RP4-813D12.3; BMP7 (margin: RP4-813D12.3; BMP7) | 5.73 | 1.55e-04 | 0.0296603652 |
|  | 12 | 9217390 | 9217769 | PDFSTATSENSEMBLUCSC | 380 | 8 | 9556 | tss | 0.0244 | 0.49245 | A2M-AS1; LINC00612 (margin: A2M-AS1; LINC00612; A2M) | 5.63 | 3.11e-04 | 0.0495606957 |
|  | 6 | 29893926 | 29894341 | PDFSTATSENSEMBLUCSC | 416 | 16 | 34757 | tss;island | 0.0218 | 0.49244 | HLA-K; HCG4B (margin: HLA-K; HLA-U; HCG4B) | 5.39 | 4.63e-07 | 0.0003195879 |
|  | 20 | 36148133 | 36149455 | PDFSTATSENSEMBLUCSC | 1323 | 41 | 25416 | gene;tss;island | 0.9773 | 0.51780 | NNAT; BLCAP (margin: NNAT; PPIAP3; RP11-425M5.5; BLCAP) | 5.21 | 1.28e-11 | 0.0000000529 |
|  | 6 | 31148332 | 31148748 | PDFSTATSENSEMBLUCSC | 417 | 16 | 35754 | island | 0.5353 | 0.96406 | POU5F1 (margin: POU5F1; PSORS1C3) | 4.98 | 3.33e-09 | 0.0000034488 |
|  | 19 | 52391234 | 52391789 | PDFSTATSENSEMBLUCSC | 556 | 10 | 20999 | tss | 0.9751 | 0.61378 | CTC-429C10.2; ZNF577; ZNF649 (margin: CTC-429C10.2; ZNF577; ZNF649) | 4.56 | 3.25e-04 | 0.0498675731 |
|  | 19 | 52391078 | 52391789 | PDFSTATSENSEMBLUCSC | 712 | 13 | 22344 | island | 0.9707 | 0.59021 | CTC-429C10.2; ZNF577; ZNF649 (margin: CTC-429C10.2; ZNF577; ZNF649) | 4.40 | 2.67e-05 | 0.0110819376 |
|  | 6 | 28058715 | 28058918 | PDFSTATSENSEMBLUCSC | 204 | 8 | 34731 | tss | 0.4956 | 0.94421 | ZNF165; ZSCAN12P1 (margin: ZNF165; ZSCAN12P1; RP1-313I6.12) | 4.37 | 1.55e-04 | 0.0296603652 |
|  | 6 | 29599012 | 29599390 | PDFSTATSENSEMBLUCSC | 379 | 10 | 33681 | gene;island | 0.5225 | 0.95166 | GABBR1 (margin: GABBR1; SUMO2P1) | 4.14 | 7.58e-05 | 0.0209443807 |
|  | 19 | 34174665 | 34175419 | PDFSTATSENSEMBLUCSC | 755 | 19 | 20688 | tss | 0.0292 | 0.42867 | CHST8 (margin: CHST8) | 3.98 | 6.75e-05 | 0.0209443807 |
|  | 6 | 32846924 | 32847845 | PDFSTATSENSEMBLUCSC | 922 | 27 | 34160 | gene;island | 0.9688 | 0.49127 | PPP1R2P1 (margin: PPP1R2P1) | 3.92 | 1.74e-05 | 0.0080291795 |
|  | 5 | 135415693 | 135416613 | PDFSTATSENSEMBLUCSC | 921 | 16 | 33146 | island | 0.8436 | 0.40933 | VTRNA2-1 (margin: VTRNA2-1) | 3.75 | 7.07e-05 | 0.0209443807 |
|  | 7 | 27183133 | 27184853 | PDFSTATSENSEMBLUCSC | 1721 | 47 | 38347 | island | 0.4520 | 0.90596 | HOXA-AS3; HOXA3; HOXA5; HOXA6 (margin: HOXA-AS2; HOXA-AS3; HOXA3; HOXA5; HOXA6; HOXA7) | 3.50 | 3.47e-11 | 0.0000000719 |
|  | 15 | 74421523 | 74421935 | PDFSTATSENSEMBLUCSC | 413 | 12 | 13789 | tss;island | 0.0586 | 0.00904 | ISLR2; RP11-247C2.2 (margin: ISLR2; RP11-247C2.2) | 3.43 | 2.74e-04 | 0.0455223165 |
|  | 20 | 13200929 | 13200992 | PDFSTATSENSEMBLUCSC | 64 | 10 | 25601 | tss | 0.0344 | 0.17401 | ISM1 (margin: ISM1) | 3.39 | 7.58e-05 | 0.0209443807 |
|  | 20 | 13200685 | 13200992 | PDFSTATSENSEMBLUCSC | 308 | 12 | 26007 | island | 0.0264 | 0.11619 | ISM1 (margin: ISM1) | 3.39 | 1.03e-04 | 0.0236793538 |
|  | 7 | 27183369 | 27184737 | PDFSTATSENSEMBLUCSC | 1369 | 41 | 37492 | tss | 0.4481 | 0.88556 | HOXA-AS3; HOXA3; HOXA5; HOXA6 (margin: HOXA-AS2; HOXA-AS3; HOXA3; HOXA5; HOXA6; HOXA7) | 3.16 | 1.30e-09 | 0.0000017973 |
|  | 13 | 25670047 | 25670238 | PDFSTATSENSEMBLUCSC | 192 | 9 | 11349 | tss | 0.5316 | 0.13619 | PABPC3 (margin: PABPC3) | 2.86 | 4.11e-05 | 0.0155042818 |
|  | 13 | 25670047 | 25670327 | PDFSTATSENSEMBLUCSC | 281 | 11 | 11609 | island | 0.5060 | 0.13619 | PABPC3 (margin: PABPC3) | 2.82 | 8.51e-05 | 0.0220396863 |
|  | 11 | 368351 | 369192 | PDFSTATSENSEMBLUCSC | 842 | 24 | 7009 | tss | 0.0338 | 0.32252 | B4GALNT4 (margin: B4GALNT4) | 2.80 | 2.74e-04 | 0.0455223165 |
|  | 18 | 12254365 | 12254690 | PDFSTATSENSEMBLUCSC | 326 | 9 | 19060 | gene | 0.0451 | 0.32332 | CIDEA (margin: RP11-64C12.9; CIDEA) | 2.77 | 1.65e-04 | 0.0296603652 |
|  | 1 | 229569608 | 229570288 | PDFSTATSENSEMBLUCSC | 681 | 14 | 4310 | island | 0.0686 | 0.31941 | ACTA1 (margin: RP5-1068B5.1; ACTA1; RP5-1068B5.3; NUP133) | 2.70 | 3.38e-04 | 0.0499764971 |
|  | 19 | 34174665 | 34175554 | PDFSTATSENSEMBLUCSC | 890 | 21 | 19974 | gene;island | 0.0292 | 0.42867 | CHST8 (margin: CHST8) | 2.67 | 9.39e-05 | 0.0229076809 |
|  | 11 | 2160540 | 2161586 | PDFSTATSENSEMBLUCSC | 1047 | 32 | 6447 | gene;tss;island | 0.0263 | 0.14572 | IGF2-AS; IGF2; INS-IGF2 (margin: IGF2-AS; IGF2; AC132217.4; INS-IGF2; MIR483) | 2.61 | 5.59e-06 | 0.0028957612 |
